# Gestural Communication and Mating Tactics in Wild Chimpanzees

**DOI:** 10.1371/journal.pone.0139683

**Published:** 2015-11-04

**Authors:** Anna Ilona Roberts, Sam George Bradley Roberts

**Affiliations:** Department of Psychology, University of Chester, Chester, Parkgate Road, Chester, United Kingdom; CNRS, FRANCE

## Abstract

The extent to which primates can flexibly adjust the production of gestural communication according to the presence and visual attention of the audience provides key insights into the social cognition underpinning gestural communication, such as an understanding of third party relationships. Gestures given in a mating context provide an ideal area for examining this flexibility, as frequently the interests of a male signaller, a female recipient and a rival male bystander conflict. Dominant chimpanzee males seek to monopolize matings, but subordinate males may use gestural communication flexibly to achieve matings despite their low rank. Here we show that the production of mating gestures in wild male East African chimpanzees (*Pan troglodytes schweunfurthii)* was influenced by a conflict of interest with females, which in turn was influenced by the presence and visual attention of rival males. When the conflict of interest was low (the rival male was present and looking away), chimpanzees used visual/ tactile gestures over auditory gestures. However, when the conflict of interest was high (the rival male was absent, or was present and looking at the signaller) chimpanzees used auditory gestures over visual/ tactile gestures. Further, the production of mating gestures was more common when the number of oestrous and non-oestrus females in the party increased, when the female was visually perceptive and when there was no wind. Females played an active role in mating behaviour, approaching for copulations more often when the number of oestrus females in the party increased and when the rival male was absent, or was present and looking away. Examining how social and ecological factors affect mating tactics in primates may thus contribute to understanding the previously unexplained reproductive success of subordinate male chimpanzees.

## Introduction

An important element in understanding the cognitive complexity underlying human language evolution is to understand the cognitive processes that govern communication in our closest living relatives [1]. Social and environmental factors are the key selection pressures which shape the cognition of any species and these selection pressures can result in the evolution of complex communication systems. One important mode of communication is gestural signalling, defined as voluntary movements of the limbs, or head and body postures, transmitted through visual, tactile or auditory channels, which appear to initiate a desired action [2]. It has been proposed that gestural communication is an important potential evolutionary precursor to human language and involves complex cognitive processes[3]. Many signals which are communicative to perceivers are not cognitively complex and may simply reflect the signaller’s internal emotional state in an involuntary way[1]. However, gestures are used flexibly, implying that signallers make voluntary choices which may be based on mental representations [1]. In this study, we focus specifically on whether individuals flexibly adjust their gestural communication[4–9] according to which other conspecifics are present or visually attending. Visual attention functions as a cue of social preferences and strength of social relationships. Understanding visual attention in a dynamic social environment requires considerable cognitive flexibility and is important for managing social relationships. Primates must not only keep track of their own relationships, but also monitor third party relationships between other group members, as changes in these relationships can have implications for their own position in the group. In certain situations, it may be advantageous for primates to use knowledge about their own and third party social relationships to adjust their gestural communication according to which social partners are present (‘audience effects’)[10]. Moreover, adjusting the production of gestures according to the visual attention of a third party audience, rather than just the presence or absence of a third party, requires a great level of social sensitivity, as the signaller has to detect and respond to cues of the audience’s visual attention[11]. Identifying how primates adjust their gestural communication according to which other conspecifics are present or looking is therefore a key factor in examining the cognitive flexibility involved in understanding third-party relationships of primates.

Examining flexibility in the use of gestures is particularly relevant in the context of the multi-male, multi-female mating systems of primates, whereby individuals employ a set of behaviours to select, attract and retain mates of the opposite sex during the fertile period of the female oestrous cycle. The evolution of primate mating strategies has been attributed both to male-male competition for mates and to female choice. The influence of these modes of sexual selection on primate mating tactics varies in intensity and can occur either independently or simultaneously. Male-male competition occurs when dominant males attempt to gain access to oestrous (sexually receptive) females and prevent subordinate males from having access to these females. Dominant males respond aggressively to the mating initiations of subordinate males, which are significantly less likely to succeed when the dominant males are nearby and perceptive[12]. When the number of competitor males is greater than the number of oestrous females, it can be more effective for a dominant male to direct aggression at the desired female, rather than attempt to actively control mating attempts by numerous subordinate males[13–17]. The dominant male can perceive mating attempts by the subordinate male and direct aggression at the female to try and prevent mating. In contexts where mating with competitively superior males confers fitness benefits to females, female choice may reinforce the advantage of male dominance rank in relation to reproductive success. The females may chose dominant males in order to reduce the risk of aggression to themselves or their offspring, or these preferences may be based on the genetic quality of the male.

As a consequence of these mating tactics, the subordinate males may attempt to shift the balance of mating competition in their favour, by employing counterstrategies[18]. An alternative way for subordinate males to gain access to females and increase their reproductive success is through modifying the gestural communication they use in mating contexts. Gestural communication achieves its goals of interaction indirectly, thus avoiding the high costs associated with contact aggression. By employing all parts of the body, gestural communication is modifiable and variable and thus provides a great deal of flexibility for the signaller[6]. For instance, individuals can modify the production of gestures, as well as the modality of the gestures, in terms of the use of auditory gestures (where sound is audible when produced), visual gestures (received by looking at the signaller) or tactile gestures (received through physical contact).

Subordinate males do not always have the physical characteristics necessary to compete directly for access to oestrous females with dominant male rivals. One important strategy that presents a solution to this problem of male-male competition is deception[19]. Gestures that communicate a signaller’s goal to the recipient can be attended to by an untargeted dominant male rival to gain a strategic advantage, in face of conflicting interests. In deception, a subordinate male deploys their usual gestural communication with the oestrous female in a way that the dominant male is unlikely to detect[20]. In line with previous definitions of deception, this can take the form of withholding information by not producing a gesture in specific contexts, as well as modifying the modality of gestural communication[19]. Dominant males monitor the mating attempts of subordinate male competitors both through their presence and also through visual attention towards the subordinate male. Understanding the visual attention and presence of the dominant male and the dominant male’s visual and acoustic perspective is therefore key in deception. Quiet visual or tactile gestures can only be perceived when the dominant male’s visual attention is directed at the signaller prior to the signal, but auditory gestures can be perceived when the dominant male is present and not looking at the signaller[6]. In deception, the subordinate male modifies the modality of the gesture to take the dominant male’s presence and visual orientation into account. Signallers may produce visual or tactile gestures when the dominant male is present to avoid auditory perception and decrease the frequency of gestures when the dominant male is looking towards the subordinate male to avoid visual perception.

Deception may also be influenced by the wider social dynamics in which the communication takes place. When party size increases, there are more oestrus and non-oestrous females for the higher-ranked male to keep track of, in order to monitor mating attempts by rival males. This is both a cognitive challenge, in tracking multiple females and their respective oestrus status, and also a physical challenge in visually monitoring multiple females and rival males in a dense forest habitat. Thus larger parties may provide more opportunities for subordinate males to employ deception and mate with oestrus females. For example, in Japanese macaques, on the days when more females were displaying mating behaviour, the average dominance rank of mating males decreased, suggesting the dominant males were less successful in disrupting the mating attempts of lower ranked males[21]. Further, in primates with larger neocortex ratios, the relationship between dominance and reproductive success in males is less strong, with lower-ranked males having more mating success[22]. Thus, in these multi-male, multi-female mating contexts, deception may provide a selective advantage in obtaining matings, and this is particularly the case for subordinate males and in parties with larger numbers of females.

In addition to these male mating tactics, oestrus females are also actively engaged in choosing particular mates. In a mating context, the visual attention of a female towards a male functions as a cue of her interest in a potential mating opportunity. Visually monitoring a potential mating partner is important for behavioural synchrony, which requires that at least one of the members of the mating dyad adjusts their behaviour to match that of the partner. If the subordinate male mating strategy is under the selection pressure of female choice, then subordinate males may be predicted to adjust their mating tactics in relation to female visual attentiveness towards male mating attempts. When the interests of the subordinate male and the oestrous female coincide, males may use visual gestures. However, if the interests of the subordinate male and the oestrous female conflict, the potential higher gain from signalling to the subordinate male, as compared to the gain to the oestrous female, suggests that the subordinate male should be willing to use more aversive gestures. Loud, auditory gestures are more aversive than visual gestures, because they are deep, sharp, sudden and high volume signals associated with high arousal that induce fear reactions in the recipients[23]. At the physiological level, loud signalling—but not visual gestures—can affect the recipient’s nervous system by increasing plasma cortisol release[24]. Loud auditory signals, as compared to visual signals, are generally more effective[25,26] and exploit the operating characteristics of the recipient’s nervous system, who are more receptive to them when exposed [27]. The coercive use of gestures may also be influenced by the social context in which the communication is taking place. The number of oestrous females in the party may influence female-female competition for access to dominant males and the dominant male’s ability to monopolize access to the oestrous females. Thus, when there are a larger number of oestrous females, the reduced female ability to gain access to dominant males may increase the chances of mating for subordinate males and make the gesturing less coercive. Further, mating gesture production may be influenced by factors that affect the level of detection and recognition of gestures in a dense forest habitat—illumination, background noise, wind, and visual access. For instance, high levels of wind and low levels of illumination may interfere with the ability of the recipient to perceive silent gestures, therefore leading to the production of more noisy gestures. High background noise may impact upon the recipient’s perception of noisy gestures, therefore leading to cessation of the signalling if the silent gesture is ineffective. Finally, ecological factors such as temperature may affect the motivation of the signaller to engage in mating behaviour. The environmental temperature influences basal metabolic rate[28] and thus an increase in temperature to the optimum temperature for mating can influence mating activity patterns[29].

There is a growing evidence for deception in the use of mating signals, in that signallers modify their vocal behaviour in relation to the risk of detection by dominant male rivals. For instance, gelada baboon females withhold copulation calls in relation to whether dominant males are nearby, which reduces the risk of punishment[30]. De Waal recounted how a young adult male chimpanzee, who was courting a female, dropped his hands over his erect penis when a dominant male was approaching[31]. However, to date little research has been carried out on the influence of female choice on mating tactics and communication in mating contexts. Until recently, systematic studies of coercion have focused almost exclusively on instances of physical aggression. For instance, the frequency of physical aggression towards females during oestrous and non-oestrous periods is associated with increased mating and reproductive success in male chimpanzees[13,14,17,32].

Chimpanzees in particular are an excellent model species to investigate the strategies underlying the use of gestures during mating initiations in primates. Chimpanzees have a varied repertoire of visual, tactile and auditory gestures which are employed in mating interactions [6,8,9]. Powerful high-ranking male chimpanzees compete for access to oestrous (sexually receptive) females more effectively than low-ranking males[12–15,33–35]. Oestrous females can mate opportunistically and promiscuously, but dominant males interfere in some mating attempts. Direct punishment of low-ranking males by higher-ranking males in the context of mating is rare—in one study, only 6.54% copulations were punished[36]. However, dominant males attempt to monopolize some oestrous females by mate guarding (the dominant male directs aggression towards an oestrous female to elicit separation from the subordinate male and to restore proximity with her), and the direct punishment of female mating with subordinate males[14]. Opportunities for mate-guarding exist because 96% of copulations occur in the presence of another male[36] and male aggression towards females is common[13]. Thus, although most aggression is directed towards females rather than other males, this aggression acts to disrupt mating by lower-ranking males[33]. Despite the fact that throughout the majority of the female oestrous cycle, the female is accompanied by the presence of a high-ranking male[12], low-ranking males are still able to achieve relatively high levels of reproductive success[37,38]. For example, in the Gombe community of chimpanzees, young, low-ranking males combined sired about 15% of the offspring, as compared to 30% of the offspring sired by the alpha male. This suggests that subordinate males are able to gain at least some matings, despite their low status and dominant males’ attempts to disrupt these matings.

Here we examine the strategies underlying the use of gestural communication during mating initiations in the Sonso community of wild East African chimpanzees (*Pan troglodytes schweinfurthii*) in Budongo Forest Reserve, Uganda. First, we detail the repertoire of mating gestures in wild chimpanzees, identifying the structural variation in signals across individuals. Second, we examine the patterns of mating gesture production and modality in relation to social and ecological factors. Finally, we explore the efficacy of the mating gestures of chimpanzees, by examining the oestrous female and the rival male responses to the gestures.

## Methods

### Study site and subjects

The Sonso community of wild, habituated East African chimpanzees (*Pan troglodytes schweinfurthii*) was observed at the Budongo Conservation Field Station, Budongo Forest Reserve in Uganda (1°35’ and 1° 55’N and 31° 08’and 31°42’ E, www.budongo.org)[39]. Budongo Forest Reserve occupies the area of 793 km^2^ at a mean attitude of 1,050 m and includes 482 km^2^ of continuous medium-altitude semi-deciduous forest. The Budongo Conservation Field Station occupies the area of a prior logging facility and is predominantly characterised by secondary forest growth, which restricts ground visibility[40]. Observations of mating gestures were conducted over period of 5 months (September 2006, April–July 2007), supplemented by observations of the socio-ecological context of mating behaviour between March and June 2008. At the beginning of the socio-ecological data collection in March 2008, the Sonso community of chimpanzees consisted of approximately 75 named individuals, 10 adult males and 22 adult females. We examined the mating initiations of six adult focal males (15 years old or older), characterised by a lack of any limb injuries, classified according to rank as either high- or low-ranking (Table A in S1 File). Focal observations were supplemented by ad libitum data collection of repertoire from two subjects (Duane and Zefa). Duane was systematically followed during 2006 and 2007, but died in 2007. The other one of the males was subject to ad libitum data collection only. Subadult males were not studied via socio-ecological samples or included in the focal analyses and were here only examined as competitors.

### Data collection protocol

Quantitative 18-minute focal animal follows were taken to examine the influence of socio-ecological conditions on the production of mating gestures. Focal subjects were chosen systematically and their behaviour recorded during a standardised observation period, attempting to sample each individual equally at different times of the day and during the study period. In order to avoid dependency in the data set, samples of the same focal subject were at least 20 minutes apart. This sampling was conservative in comparison to previous studies, which have established that sampling chimpanzee behaviour every 15 minutes was independent, e.g. [41]. Behavioural data collected in this study came from three sources. First, we recorded gestural behaviour of focal individuals. A digital video camera recorder (SONY DCR HC32E) recorded mating gestures continuously, with the camera centred on the focal animal but also taking a wider view to include interactants within the visible presence of the focal individual. Second, we recorded submissive behaviour (vocalisations, gestures, fleeing), from focal subjects during focal follows and *ad libitum* from other adult males in the community. Third, 18-minute instantaneous sampling of association patterns and ecology (samples) accompanied data collection of gestures. These consisted of 9 scans (subsamples) at 2 minute intervals of the focal bodily orientation and party composition. The subsamples recorded the identity and reproductive status (if female) of adult individuals present in the party; the identity of the highest ranking male (rival male) in the audience and the nearest neighbour, including their proximity, bodily orientation and visual access to the focal male. Also during the 18-minute sample, 3 scans at 6 minute intervals recorded ecological variables (ambient noise, illumination). The temperature and wind were recorded at the end of the sample. The recording of ecology at 6, 12 and 18 minutes was aligned with the 9 subsamples at 2 minute intervals.

For the party composition scans, we recorded the identity of all adult individuals who were present in the same party. The party was defined as the group of individuals showing coordination in behaviour (e.g. chorus), within a spread of about 35m. Female chimpanzees exhibit sexual swellings, which are enlarged areas of the perineal skin that vary in size over the course of the menstrual cycle. The largest swelling is observed during the reproductively fertile period. For all reproductively mature (exhibiting menstrual cycle and observed mating) females in the party, reproductive status was recorded based on swelling size and rated on a scale from 1–4, with the maximum tumescence scored 4.

We considered the influence of the audience by recording the identity of the highest ranking male (adult or subadult) in the audience (‘rival male’). We also recorded the identity of the individual (adult or subadult) nearest to the focal male (‘nearest neighbour’). The bodily orientation of the focal male towards the nearest neighbour, and towards the rival male (and vice versa) was recorded as visually oriented towards (one had the other within their field of view, up to 45 degrees body turn) or away (facing another with the back). The visual access between the focal male and the nearest neighbour, and the focal male and the rival male, was recorded as good (possible two way visual exchange through unobstructed visual channel), medium (signaller and recipient can only see certain body parts of each other, visual channel obstructed to certain degree) and poor (difficult two way visual exchange, visual channel obstructed).

We considered the influence of ecological variables on the propensity to gesture by recording illumination, ambient noise, temperature and wind. The level of illumination by natural light source was measured using a Tizaro 4 in 1 Environmental Meter (measuring range 0–20000 Lux), positioned approximately 1 m above the ground, pointing upward towards the canopy, away from patches of direct sunlight. The level of illumination was measured for 5 s to obtain maximum value, moving the device clockwise by 180° to obtain one measurement from each of the two cardinal directions (West and East). A mean of these two measurements was calculated. All ambient noise measurements were recorded with a Precision Gold NO5CC sound level meter (measuring frequency range 31.5Hz–8KHz), held approximately 1 m above the ground. A time constant of 1 second and a ‘C’ frequency weighting was used. This frequency weighting was chosen because auditory gestures are of low frequency and therefore the ambient noise in the frequency range of the auditory gestures is crucial for production of gestural communication. The ambient noise was measured by aligning the sound level meter horizontally to the ground with one of the four cardinal directions. The ambient noise level was measured for 5 s, moving the device clockwise by 90° to obtain one measurement from each cardinal direction. The mean of these four measurements was then calculated. We recorded temperature at ground level, in one direction using an environmental meter (Tizaro 4 in 1 Environmental Meter). The wind speed was recorded in meters per second using a Tizaro 4 in 1 Environmental Meter, facing the air flow sensor to the source of wind for 20 seconds. The instantaneous sampling of association patterns and ecology was conducted by two experienced field assistants, who were unaware of the aims of the study and did not collect any data on gestural communication. These field assistants undergo an inter-observer reliability test annually, with results consistently above 0.85 Spearman’s rank correlation coefficient, *r*_s_. The video recording of the gestures was carried out by the author (AR) and thus the socio-ecology and gestural data were collected independently of each other.

### Data coding

Using video recordings, non-verbal behaviour was scored as gestural communication if it was a movement of the limbs, body, head or locomotory gait that was intentional and communicative[6]. Gestures were scored as intentional when: 1) the signaller directed a gesture at a recipient and monitored the recipient’s response during and after the gesture, 2) the production of a gesture was sensitive to the recipient’s visual attention state, 3) there was persistence in gesture production when the recipient failed to respond, 4) a gesture induced change in recipient’s behaviour by non-mechanical means. We evaluated these criteria for each gesture type separately, using pooled data across subjects[6]. Gestures above the threshold of 60% of cases were classified as intentional. A description of gesture types and corresponding video clips can be found in [6,9]. Gesture events were assigned to broad morphological categories (e.g. head, leg, manual) to distinguish between single gestures and their combinations (i.e. more than one gesture type made simultaneously by the signaller). Gestures were scored in accordance to whether they were uni or multimodal (accompanied by simultaneous production of vocal behaviour or facial expressions). The modality of gesturing was classified as either visual/ tactile (gesture reception by the recipient was possible by looking at signaller or via physical touch) or auditory (gesture reception by the recipient was possible by listening to the gesture, without visual contact). Gestures were classified in relation to whether they occurred in sequences of single or multiple gesture events (more than one gesture made consecutively by one individual towards the same recipient, and with the same goal, within a maximum of 30 s interval). A bout was defined as a series of sequences of either single or multiple gestures, produced towards the same female, with the same goal (copulation) during the same sample. Mating gestures were identified from context, i.e. a gesture was scored as a mating initiation if it was accompanied by penile erection, directed towards a fully tumescent female, and elicited approach for mating, or persistence in gestural communication if an approach was not made[34,42]. To examine mating gestures, for each gesture event following data was coded: 1) Signaller—defined as the individual performing a gesture; 2) Recipient—individual at whom the gesture was most clearly directed, as identified from the orientation of head and body of the signaller during or immediately after performing a gesture. The signaller had the recipient within its field of view (up to 45 degrees body turn); 3) Response—categorised as change in the recipient’s behaviour following a gesture (response present) or response absent. Response present was further classified as a) redirecting the recipient’s visual attention state towards the signaller, b) approach followed by non-interactive behaviour (e.g. resting, feeding), c) approach followed by copulation (with/ without copulation call), d) other; 4) Visual attention of signaller and recipient prior, during and after the gesture. Attention present was recorded when one had the other within their field of view (up to 45 degrees body turn), otherwise attention was recorded as absent; 5) Rival reaction, defined as the rival directing behaviour towards the signaller within 1 minute of the first gesture in the bout. A rival reaction was scored if a rival male performed an approach, threat or physical aggression, which elicited pant-grunts, submissive gestures, retaliation or cessation of mating attempts in the focal male. The video was coded by an experienced observer and the inter-observer reliability test of the video footage by an independent coder reliably assigned gesture events to the categories of gesture type (K = 0.76) and response type (K = 0.86).

### Classification of variables

For each subsample at 2 minute intervals, the presence or absence of a mating initiation gestures were scored. To ensure independence, the gestures were scored as present if they occurred as the first gesture within the 2-minute interval (rather than continuation of sequences from previous 2-minute interval). For each interval, the dominant gesture modality was scored, prioritising the auditory modality over the visual/ tactile modality if both modalities were present. Moreover, for each first gesture within the gesture bout, we noted the presence or absence of copulation. The female’s reproductive status was identified as oestrous (swelling 3–4) or non-oestrus (swelling 0–2) and the total number of oestrous and non-oestrus females present in the party was recorded. Visual access was categorised as good (good and medium combined) or poor. Since the values of ambient noise, illumination, wind and temperature were correlated with the values obtained at preceding intervals (if taken every two minutes), we extrapolated values obtained at 6 minute intervals to the preceding 6 minutes (illumination, ambient noise) or 18 minutes (temperature, wind). The dominance ranks of males were determined from the directionality and frequency of submissive behaviour. An individual produces submissive behaviour in response to an approach or aggressive act from another individual. Submissive behaviour includes submissive gestures (such as crouching, bobbing), vocalisations (pant-grunt, scream) and fleeing, which function as a sign of subordinance in chimpanzees[43]. We summed the number of submissive behaviours for each pair of males and created a dominance matrix, such that the number of interactions below the diagonal of the matrix was as high as possible[43]. We only considered ranks of adult individuals. The males were classified as adult (15 years old or older) or subadult (11 to 14 years old). When considering the relative ranks of the rival male to the focal male, the ranks were used to score the rival male as either lower or higher in the hierarchy (Table B in S1 File). Focal males were classified as either high- or low-ranking, using a median split (Table A in S1 File). Subadult males were always considered subordinate to adult males.

### Statistical analyses

As each gesture could not be considered an independent data point, we used generalized linear mixed models (GLMM) to examine the factors influencing the production of mating gestures, the modality of mating gestures and the presence or absence of copulations. In the GLMM models, the data on gestures were hierarchically structured with three levels—Level 1 was the focal individual, Level 2 was the 18 minute sample and Level 3 was the 2 minute subsample. GLMM is a modified form of regression analysis designed to deal with data with a hierarchical clustering structure. All the GLMM models had binary outcome variables–the presence or absence of a gesture, modality (auditory or visual/tactile) or the presence or absence of copulation. Thus, as with logistic regression models, these GLMM models included data both where the event of interest was present and also where it was absent. The sample size for each model was therefore not just limited to when the event of interest took place. The absence of behaviour in the 2 minute sub-sample was the reference category (with visual/ tactile the reference category for modality) and the models were fitted using a binomial error structure with logit link. Mating gestures were present in 37 of the 935 sub-samples available for analysis (3.95%). Of the 37 mating gestures, 8 were visual/ tactile (21.6%) and 29 were auditory (78.4%). Copulations were considered only when mating gestures were present. Of the data available for analysis, copulations were present in the 11 of the 23 bouts of gestures (47.8%). A full list of all the predictor variables considered in the models is given in Table 1. All gesture types observed in this study are outlined in Table 2. The random effects included were the focal male identity and the focal male identity by sample number–for these effects random intercepts were used. Random slopes were not used in this model, as the key focus was on how the predictor variables influenced the presence or absence of behaviour, rather than how the effect of these variables differed between subjects. For the tests of fixed effects and coefficients, robust estimation was used due to the small sample size. The penalized quasi-likelihood (PQL) algorithm was used to estimate the GLMMs. Contingency tables for Models 1–6 can be found in Table D in S1 File. For all statistical tests, the alpha level was set at 0.05 and all tests were two-tailed. The Generalized Linear Model function in IBM SPSS Statistics 21 was used for all the models.

**Table 1:** Predictor variables included in generalised linear models predicting the presence or absence of a mating gesture.

Predictor variable	Definition	Frequencies, Mean±SD and median (quartiles)
**Ecological variables**		
Noise	Noise level (dB)	57.89±23.33 53.35 (51.10, 56.40)
Illumination (log)	Illumination level (lx)	2.67±0.59 2.62 (2.26, 3.04)
Temperature	Temperature (°C)	25.41±2.65 25.50 (23.10, 26.90)
Wind	Wind (0 = no wind, 1 = wind)	0 = 897 1 = 29
**Social variables**		
Focal rank	Rank of focal male (0 = low, 1 = high)	0 = 691 1 = 244
Non-oestrus females	Number of non-oestrus females in party	0.59±0.86 0.00 (0.00–1.00)
Adult males	Number of adult males in party	2.84±1.81 3.00 (1.00–4.00)
Oestrus females	Number of oestrus females in party	1.13±0.59 1.00 (1.00–1.00)
Adult rival presence	Age of the rival (0 = subadult, 1 = adult)	0 = 94 1 = 836
Rival relative rank	Rank of rival male relative to rank of the focal (0 = lower, 1 = higher)	0 = 213 1 = 657
Rival proximity	Proximity of focal to rival male (m)	12.13±9.83 10.00 (4.00–20.00)
Rival orientation to focal	Orientation of rival male towards focal (0 = oriented away from focal, 1 = oriented towards focal)	0 = 268 1 = 591
Focal orientation to rival	Orientation of signaller towards rival (0 = rival not in signaller’s view, 1 = rival in signaller’s view)	0 = 252 1 = 607
Rival visual access	Visual access between rival and focal male (0 = poor visual access, 1 = good or medium access)	0 = 243 1 = 620
Nearest oestrous female	Whether oestrous female is nearest neighbour to focal (0 = no, 1 = yes)	0 = 742 1 = 193
**Nearest neighbour (oestrus female) variables**		
Nearest neighbour proximity	Proximity of nearest neighbour to focal male (m)	6.42±6.02 4.00 (2.00–10.00)
Nearest neighbour orientation to focal	Orientation of nearest neighbour towards signaller (0 = oriented away from signaller, 1 = oriented towards signaller)	0 = 63 1 = 129
Focal orientation to nearest neighbour	Orientation of signaller towards nearest neighbour (0 = nearest neighbour not in signallers view, 1 = nearest neighbour in signallers view)	0 = 43 1 = 149
Nearest neighbour visual access	Visual access between focal male and nearest neighbour (0 = poor visual access, 1 = good or medium visual access)	0 = 11 1 = 182

Due to missing data, the total number of cases differs between variables

**Table 2:** Repertoire of mating gestures by individual males.

Gesture type/ Subject^1^ ^,^ ^2^	Bwoba	Hawa	Kato	Musa	Nick	Squibs	Duane	Zefa
Arm beckon^V^					x			
Arm raise^V^		x						
Bounce^A^		x	x	x				
Clip by hand^A^		x	x					
Clip by mouth^A^		x	x					
Cupped extend^V^			x					
Drag object^V^								x
Forceful extend^V^			x					
Hang^V^							x	
Hit object^A^			x					
Hold object^V^			x					
Inspect^T^		x						
Jump^V^		x						
Linear sweep^V^		x						
Poke^T^		x						
Present genitals^V^	x	x	x	x	x	x	x	
Pull another^T^	x							
Shake mobile^A^		x						
Shake stationary^A^	x	x	x	x			x	
Stamp quadrupedal^A^		x						x
Stamp sitting^A^		x	x	x			x	
Stationary stiff^V^		x						
Strip leaf^V^		x						
Stroke short^T^		x						
Sway^V^		x	x					
Touch self^V^		x						
Turn back^V^			x					
Unilateral swing^V^			x		x			
Vertical extend^V^		x			x			
Walk stiff^V^		x			x			
Wipe^V^		x						
Total number of gesture types/ subject	3	21	13	4	5	1	4	2

^1^ Description of gesture types and video clips showing all gestures can be found in Roberts, A. I., Roberts, S. G. B. & Vick, S.-J. The repertoire and intentionality of gestural communication in wild chimpanzees. *Animal Cognition* **17**, 317–336 (2014) and also Roberts AI, Vick S-J, Roberts SGB, Buchanan-Smith HM, Zuberbühler K (2012) A structure-based repertoire of manual gestures in wild chimpanzees: statistical analyses of a graded communication system. Evolution and Human Behavior 33: 578–589.

^2^Observations of repertoire include focal and ad libitum observations of focal and non-focal (Duane and Zefa) subjects

^A^Auditory gesture

^V^Visual gesture

^T^Tactile gesture

As all the predictor variables could be related to the presence or absence of a mating gesture, initially a full model was created by entering all variables into the GLMM (Table 3). In a smaller number (*n* = 193) of sub-samples, the nearest neighbour when the focal male was gesturing was an oestrous female. In these samples, additional variables relating to the nearest neighbour were available for analysis (Table 4). To examine the effect of these nearest neighbour variables on the presence or absence of mating gestures, a second GLMM was produced (Model 2), retaining the significant variables from the full model relating to orientation of the rival male and including the additional nearest neighbour variables. Of this reduced data set, mating gestures were present in 17 of the 193 sub-samples (8.8%). Visual access to the rival male and to the nearest neighbour were related to each other–when visual access to the rival male was good, visual access was significantly more likely to be good to the nearest neighbour oestrous female (χ^2^ = 18.89, df = 1, p < 0.001). Because these two variables co-varied, in Model 3 visual access to the rival male was excluded, and visual access to the oestrous female included.

**Table 3:** Models 1, 4, 6: Influence of rival presence, visual attention and other predictor variables on production/ modality of gestures and copulations.

Predictor variable	Coefficient estimate	Standard error	*t*	p
Model 1. Response variable: Presence or absence of gesture (Overall percentage of cases assigned correct = 96.7%)				
*Fixed effects*				
Noise	-0.000	0.002	-0.144	0.885
Illumination	-0.039	0.362	-0.108	0.914
Temperature	-0.188	0.155	-1.217	0.224
Wind	13.020	1.177	11.065	<0.001***
Focal rank	0.119	1.332	0.089	0.929
Non-oestrus females	0.622	0.318	1.955	0.051
Adult males	-0.004	0.198	-0.020	0.984
Oestrus females	0.886	0.245	3.617	<0.001***
Adult rival presence	2.933	1.310	2.239	0.025*
Rival relative rank	-2.487	1.217	-2.044	0.041*
Rival proximity	0.018	0.027	0.678	0.498
Rival orientation to focal	0.972	0.469	2.073	0.038*
Focal orientation to rival	0.361	0.274	1.316	0.188
Rival visual access	-1.684	0.240	-7.004	<0.001***
Nearest oestrous female	-1.584	0.379	-4.174	<0.001***
*Covariance parameters*				
Focal male	1.629	2.190	-	0.457
Focal male x sample number	1.155	0.666	-	0.083
Model 4. Response variable: Auditory gesture or visual/ tactile combined (Overall percentage of cases assigned correct = 100%)				
*Fixed effects*				
Oestrus females	19.507	0.912	21.394	<0.001***
Adult rival presence	40.325	0.847	47.586	<0.001***
Rival relative rank	-37.840	0.238	-159.113	<0.001***
Rival orientation to focal	-1.029	0.478	-2.154	0.043*
Rival visual access	-0.748	0.525	-1.425	0.169
Nearest oestrous female	20.142	0.543	37.124	<0.001***
*Covariance parameters*				
Focal male	5.402	13.047	-	0.679
Focal male x sample number	2.817	5.800	-	0.628
Model 6. Response variable: Presence or absence of copulation (Overall percentage of cases assigned correct = 95%)				
*Fixed effects*				
Oestrus females	1.247	0.573	2.174	0.049*
Adult rival presence	38.876	0.163	238.428	< 0.001***
Rival relative rank	-20.477	0.383	-53.424	< 0.001***
Rival orientation to focal	2.361	0.704	3.354	0.005**
Rival visual access	-18.380	0.457	-40.191	< 0.001***
Nearest oestrous female	-1.571	0.738	-2.128	0.053
*Covariance parameters*				
Focal male	3.993	6.821	-	0.558
Focal male x sample number	1.605	3.135	-	0.609

*p < 0.05

**p < 0.01

***p < 0.001

For dichotomous variables the odds ratio for a value of zero is given.

See Table 1 for definition and descriptive data for predictor variables included in these Models.

**Table 4:** Models 2, 5: Influence of oestrous female’s and rival male’s visual attention and other predictor variables on production/ modality of gestures.

Predictor variable	Coefficient estimate	Standard error	*t*	p
Model 2. Response variable: Presence or absence of gesture (Overall percentage of cases assigned correct = 95%)				
*Fixed effects*				
Wind	19.185	0.559	34.347	<0.001s**
Oestrus females	-0.572	0.615	-0.930	0.354
Rival orientation to focal	1.778	0.582	3.058	0.003*
Rival visual access	-18.678	0.249	-74.878	<0.001**
Nearest neighbour proximity	-0.154	0.038	-4.066	<0.001**
Nearest neighbour orientation to focal	-1.118	0.406	-2.758	0.007*
Focal orientation to nearest neighbour	-1.758	0.328	-5.363	<0.001**
*Covariance parameters*				
Focal male	0.975	1.389	-	0.483
Focal male x sample number	1.330	1.217	-	0.275
Model 5. Response variable: Auditory gesture or visual/ tactile combined (Overall percentage of cases assigned correct = 100%)				
*Fixed effects*				
Oestrus females	1.108	1.585	0.699	0.505
Rival orientation to focal	-0.742	0.309	-2.400	0.043*
Nearest neighbour proximity	-0.320	0.108	-2.960	0.018*
Nearest neighbour orientation to focal	30.689	1.036	29.631	<0.001**
Focal orientation to nearest neighbour	2.551	1.707	1.494	0.173
*Covariance parameters*				
Focal male	11.240	18.966	-	0.553
Focal male x sample number	3.963	7.917	-	0.617

*p < 0.05

**p < 0.001

For dichotomous variables the odds ratio for a value of zero is given.

See Table 1 for definition and descriptive data for predictor variables included in these Models.

For the models relating to the gesture modality (auditory or visual/tactile), a similar approach was followed. The initial models were produced including just the significant variables from Model 1. For the sub-samples when the nearest neighbour was an oestrous female, an additional model was produced which included the nearest neighbour variables. For the model relating to copulations, this additional nearest neighbour model was not produced as the key issue was how the presence and absence of copulations was influenced by variables relating to rival male, rather than the oestrous female. These models relating to gesture modality and copulations were based on the sub-samples when mating gestures were present and thus necessarily had a smaller sample size than Models 1–3 predicting the presence and absence of gestures.

For all models, potential issues of collinearity between the predictor variables were addressed by examining the Variance Inflation Factors (VIF) derived from a multiple regression, with the same binary dependent variable as the GLMM models. VIF values of above 10 indicate potential collinearity issues. This approach of using VIF to examine collinearity has previously been used in relation to GLMM models with a binomial response variable, examining the presence or absence of alarm calling in wild chimpanzees[44]. For all models, the VIF values did not indicate any collinearity issues, with the largest VIF 5.863. In all non-parametric analyses, we avoided pseudoreplication by using the individual rather than the gesture as the unit of analyses, combining observations on focal and non-focal subjects for analyses (N = 8). We calculated individual frequencies and converted these into proportions for each individual for each behavioural category (gesture auditory or visual/tactile; recipient looking or not looking at the signaller prior to the gesture). We used individual proportions because the frequencies of gestures differed between individuals.

## Results

### The repertoire and structure of mating gestures

The focal chimpanzees produced mating gestures at a median rate of 2.60 gestures per hour (IQ = 0.62–7.78), and displayed a total of 31 gesture types to initiate mating (Table 2). The median number of mating gesture types per focal male was 4.5 (IQ = 2.5–15.00). Gestures occurred as either single or combined gesture events. The median percentage of gestures occurring as a single gesture was 57.4 (IQ = 40.42–91.68), whilst the median percentage of gestures occurring as a combination of gesture types was 42.60 (IQ = 8.33–59.58). There was no significant difference between the percentage of gestures occurring as a single gesture, as compared to occurring as a combination of gesture types (Wilcoxon signed ranks test, *T* = 9, *n* = 7 (1 tie), *p* = 0.47). The median percentage of gestures occurring as unimodal gestures and multimodal gestures was also examined. Significantly more gestures occurred as unimodal gestures (median percentage = 100.00, IQ = 98.80–100.00) as compared to multimodal gestures (median percentage = 0.00, IQ = 0.00–1.20; *T* = 0, *n* = 8, *p* = 0.01). Across the individual males, there was no significant difference in the proportion of auditory gestures produced (median 0.45, IQ = 0.00–0.57), as compared to visual/tactile gestures (median 0.61, IQ = 0.43–1.00; *T* = 7, *n* = 7, *p* = 0.53). Mating initiations occurred in bouts of either single or multiple sequences of gestures. Overall, 23 bouts of mating gestures were recorded, of which 3 were composed of a single sequence of gestures, and 20 were composed of multiple sequences of gestures. The median number of sequences in a bout was 3.00 (IQ = 2.00–5.00) and the median number of gesture events (single and combined) within a sequence was 1 (IQ = 1.00–1.00).

### Influences on presence or absence of mating gestures

The sample sizes for the number of gestures, gesture modality and copulations for each of the six focal males are presented in Table A in S1 File. The description of all the predictor variables considered in the models is given in Table 1. The focal males were observed in the presence of rival males and oestrous females, with the highest ranking male in the audience labelled a ‘rival male’. The presence or absence of mating gestures was significantly affected by the presence of an adult rival male, the rival male’s visual orientation and also the degree of visual access between the focal male and the rival male (Model 1, Table 3). Focal males were more likely to produce a mating gesture when an adult rival male was not present in the party. Focal males were also more likely to produce a mating gesture when the rival male was oriented away from them, when they had good visual access to the rival and when the rival male was higher-ranking than the focal male. In terms of oestrous females, mating gestures were significantly more likely to be produced by the focal male as the number of oestrous females in the party increased and if the nearest neighbour was an oestrous female. Finally, mating gestures were more likely to be produced when there was no wind.

When the individual nearest the focal male was the oestrous female, all mating gestures were directed at her. We therefore examined the behaviour of focal males when the oestrous female was the nearest neighbour, labelling her the recipient of the gesture. The nearest neighbour variables relating to oestrous females also significantly influenced the presence or absence of mating gestures (Model 2, Table 4). Mating gestures were significantly more likely when the nearest neighbour was in closer proximity to the focal male, was oriented towards the male and the male was oriented towards the nearest neighbour. Finally, mating gestures were significantly more likely when the visual access between the focal male and the nearest neighbour was good (Model 3, Table C in S1 File).

### Influences on the modality of mating gestures

We used two GLMM models to examine the variables that influenced the modality of mating gestures. The response variable for both models was whether the gestures were auditory or visual/tactile, with visual/tactile gestures as the reference category. Focal males were significantly more likely to produce an auditory gesture, as opposed to a visual/tactile gesture, when the adult rival male was not present, when the rival male was higher-ranking than the focal male, when the rival male was oriented towards the focal male and when the nearest neighbour was a non-oestrus female (Model 4, Table 3). We examined the modality of gesturing when the oestrous female was the nearest neighbour, labelling her the recipient of the gesture. Focal males were significantly more likely to produce an auditory gesture when the nearest neighbour was oriented away from the focal male and the nearest neighbour was in closer proximity to the focal male (Model 5, Table 4).

### Rival male and oestrous female reactions to mating gestures

Out of the nine instances of male gestures when the higher-ranking dominant male was present and looking at the subordinate male, only two instances of aggression to the subordinate male were observed within the first minute of production of the first gesture in the sequence (22.2% of instances). In terms of oestrous female reactions, following the production of a sequence of gestures, the dominant response type—approach for copulation–occurred in a median proportion of 0.28 of cases (IQ = 0.12–0.75). In the cases where an approach for copulation did not occur, other responses related to approaching or monitoring soliciting males were present: approach signaller (median = 0.25, IQ = 0.00–0.44) and less commonly look at signaller (median = 0.00, IQ = 0.00–0.07). In a median proportion of 0.20 cases, there was no response (IQ = 0.00–0.61). Bouts of gestures were more successful in eliciting copulations, with 47.8% of bouts leading to copulation, as compared to 28% of individual sequences leading to copulation. The median number of sequences within a bout that led to copulation was 2.00 (IQ = 2.00–5.00), with a median duration from the first gesture in a bout to copulation of 88.00 seconds (IQ 19.00–305.00). Of all the bouts of gestures that led to copulation, none of the bouts contained additional gestures produced by the focal male towards the same oestrous female within two minutes of copulation. Of the 11 instances of copulations observed, 3 copulations were accompanied by a copulation call. Of these three copulations, an adult rival male was present for two of the copulations and absent for the other copulation.

We examined instances of copulations between the focal male and the oestrous female by assigning the presence or absence of copulation to each first gesture in a bout (*N* = 23). In total, 11 separate instances of copulation were preceded by gestures. We used a GLMM model to examine the factors that influenced the success of the focal male in eliciting copulation from the female (Model 6, Table 3). The binary outcome variable in this model was whether or not copulation took place following the first gesture in a series of sequences by the focal male. Gestures were significantly more likely to elicit copulations when the adult rival male was absent and when the rival male was higher-ranking than the focal subject. Gestures were also significantly more likely to elicit copulations when the rival male was oriented away from the focal male, when visual access between the focal male and the rival male was good and when there were more oestrous females in the party.

We also examined how the presence of a response to the gestures by an oestrous female was influenced by visual attention of the oestrous female towards the focal male. The median proportion of gestures eliciting a response from the female was significantly higher when the female was oriented towards the focal male prior to the gesture (median = 1.00, IQ = 0.97–1.00), as compared to when the female was oriented away from the focal male (median = 0.00, IQ 0.00–0.03, *T* = 0, *n* = 6, *p* = 0.03).

## Discussion

Our results suggest that chimpanzees modified the production and modality of their gestures, according to the conflict of interest with the female, which in turn was influenced by the higher-ranking male’s visual orientation and presence[45]. When the higher-ranking rival male was oriented away from the focal male, the focal male was more likely to use visual/tactile gestures than auditory gestures. Here the interests of the focal male and the oestrous female coincide, as the female is not at risk of aggression from either the focal male, or the higher-ranking rival male. In this context, the use of visual and tactile gestures marked a mutual mating preference between the focal male and the female, because it is in the female’s interests to mate with multiple males to avoid the risk of infanticide[17]. However, in other contexts, the focal males’ and the females’ interests’ conflict and the gestures were more coercive[45]. When the adult rival male was absent, the focal male was more likely to use auditory gestures than visual/tactile gestures. Here there is a conflict of interest between the focal male and the female, because the focal male wants to elicit mating, but the female is at risk of aggression from the focal male in the absence of the rival adult male[13,14]. Further, when the higher-ranking rival male was present and was oriented towards the focal male, the focal male was also more likely to use auditory gestures rather than visual/tactile gestures. In this situation, the interests of the focal male and oestrus female conflict because the focal male wants to elicit mating, whereas the direction of visual attention by the rival male suggests that he is alert to the focal male’s mating attempts. Moreover, the female may have a mutual preference in mating with the higher-ranking male and so may be less likely to respond to a visual/tactile gesture[14]. This flexible use of gestures was an effective mating strategy, in that male gesturing was more likely to elicit copulation when the dominant male was absent (coercive tactic) or was present and oriented away from the focal male (non-coercive tactic). This use of gestural communication to achieve opportunistic mating in the presence of rival males may thus contribute to the relatively high reproductive success of low-ranking chimpanzee males[37].

However, the production of gestures was reduced when the higher-ranking male rival was present and looking towards the signaller. One explanation for this result is that the production of gestures was directly influenced by the risk of aggression from the higher-ranking male towards the subordinate male. Although direct aggression towards males in mating contexts is rare in chimpanzees[36], the visual attention of the higher-ranking rival towards the lower-ranking focal male may be sufficient to interfere in subdominant male mating attempts[46]. Alternatively, the production of mating gestures may be reduced when the rival male was present and looking at the signaller because in these contexts, females were less likely to approach the signaller for copulation. The fact that the low-ranking males used auditory gestures and not visual gestures when the rival male was looking at the signaller suggests that the overall reduction in the production of mating gestures in this context may be due to the inefficiency of gestures in eliciting mating, rather than the risk of aggression or the low ranking male trying to deceive the higher ranked male and avoid detection.

Additionally, wider social dynamics also influenced the propensity of males to engage in signalling. The number of non-oestrus females in the audience had an almost significant positive effect on the frequency of gesturing (*p* = 0.051). The number of non-oestrus females in the party was positively correlated with the number of oestrous females in the party. It may become increasingly difficult for a higher-ranking rival male to monitor females in parties of increasing size, and keep track of which females are sexually receptive. The non-oestrus females were sometimes gestured to, but not copulated with. The inability of the higher-ranking rival male to monitor all females simultaneously may have created mating opportunities for the lower-ranking males and made gesturing more effective. Lower-ranking males produced more mating gestures when the number of oestrous females in the party increased. Gestures were also more likely to elicit copulation when the total number of the oestrous females in the party was higher. However, the increase in the rate of auditory gestures in parties of increasing size suggests that the matings were coercive, and that females had reduced mating opportunities with preferred partners when the number of oestrous females was higher. These findings support previous research which suggests that the use of conspicuous signals of oestrous state by female chimpanzees (such as swelling size) may have arisen to increase female conspicuousness to desired males and to reduce mating harassment by subordinates[47].

Male mating tactics notwithstanding, it is the females, not the males, who are more likely to be the target of reactions by the rival males[14]. Females were more likely to approach for copulation when the rival male was absent, or was present and oriented away from the focal male, suggesting flexibility in female behaviour in relation to the rival male’s visual attention and presence. It could be argued that the flexible female responses to the gestures were driven by associative learning or an inferential process, in that females learnt if they respond to visual or tactile gesture when the rival male is not oriented towards the male, they would not be punished[48,49]. The females may have thus responded to the gestures based on observable characteristics of the situation, without any awareness of the higher-ranking male’s mental state [48,50–52]. Alternatively, female responses could be based on their awareness of the visual and acoustic perspective of the higher-ranking rival male[53]. Finally, female responses could be driven by the characteristics of the gesture itself[26]. Previous research has suggested that females may be driven to respond to more conspicuous signals without much flexibility to modify their behaviour [27]. In our study, females were more likely to approach for copulation when the male used auditory gestures in the absence of the adult rival male. This suggests that females were less responsive to visual and tactile gestures than auditory gestures when there was a conflict of interest. On the other hand, a lack of responsiveness to the auditory gestures when the rival male was present and looking at the focal male may suggest that the use of auditory gestures did not influence the female to inadvertently produce an approach for copulation. As the female is monitored by the higher-ranking rival male, she flexibly abstained from approaching in response to the auditory gestures of the lower-ranking focal male when the higher-ranking rival was attentive. Thus, females play an active role in mating behaviour[54] and female responses are flexibly tailored to reduce the risk of aggression and to maximise female choice.

Finally, our results suggest that characteristics of the ecological environment are also important in shaping gesture use in chimpanzees. When wind intensity increased, the production of mating gestures decreased. One possible explanation for this result is that when wind intensity increased, the ambient temperature decreased, thus reducing the likelihood of the production of mating gestures, as other activities such as feeding or grooming took priority in the cold temperature. However, in this study, the temperature and wind intensity were not correlated and the temperature did not seem to have an influence on the frequency of gesture production. Alternatively, the relationship between wind intensity and the frequency of mating gestures suggests that the degree of difficulty of signal transmission in a forest habitat influenced communication. It has long been established that animal signals evolve to function effectively in a particular signalling habitat[55]. Signallers play an important part in this process, by choosing conditions that are conducive to reliable transmission and detection of the signals. The influence of wind intensity and motion noise on the production of gestures of chimpanzees living in the forest habitats may be important because wind causes foliage and branches to sway in both the horizontal and vertical planes[56]. Many chimpanzee mating gestures incorporate in their display swaying movement in both the horizontal and vertical planes and use objects from the environment (e.g. leaves, branches). The influence of wind action on the foliage and branches may therefore influence gesture transmission and detection by obscuring and imitating such gestures. Previous studies on the influence of wind on the production of courtship displays showed that Jacky lizards modify the structure of their visual display when the environmental motion noise influenced by wind increases[57]. The reduced production of mating gestures when the wind is stronger thus suggests that chimpanzees are sensitive to ecological factors that may influence the efficiency of their signalling. One limitations of our study is that only 4% of the 935 2-minute subsamples contained mating gestures, and the largest sample size for both gestures and observation hours was for the two lowest ranking and youngest adult individuals. This sample size and unequal sampling may have influenced the results and thus our conclusions should be treated as preliminary until further studies of mating tactics and communication are carried out.

To conclude, in this study we have shown that male mating tactics vary according to the characteristics of the social audience and the environment. Focal males are more likely to produce visual and tactile gestures when there is no conflict of interest with the oestrous female and are more likely to produce auditory gestures when there is a conflict of interest with the oestrous female. This conflict of interest is influenced by the dominant male’s presence and visual attention. The production of mating gestures is also influenced by the number of oestrous and non-oestrus females in the party and the intensity of wind. Female chimpanzees play an active role in shaping male mating tactics by responding more often when the dominant male is absent, or is present and looking away, and when the number of oestrous females in the party is higher. Future studies could further explore how the signaller’s continued efforts at signalling, and the female responsiveness to mating gestures, is affected by the orientation of both the signaller and other conspecifics, as well as the rival male responsiveness to the focal male mating attempts. For instance, future studies could examine if the female may be cued to approach and copulate with the signaller when the rival male does not respond to the signaller’s efforts, but is perceptive of the gesture. This approach moves away from an information-theoretic view of communication, which focuses on the information transmitted from the signaller (the focal male who communicates to elicit mating) to the recipient (female). In contrast, a dynamic systems paradigm[58] focuses on the meaning created by an interaction between the signaller and recipient in a particular context. In the chimpanzee mating context, the response of the recipient, and outcome of the interaction, is dynamically dependent not just on the information that is transmitted in the gesture, but also the dyadic interaction between the male and the female and the broader socio-ecological context–notably the presence and visual orientation of other males and females which influence the possibilities for opportunistic mating[58].

## Supporting Information

S1 File S1 file contains following tables: Table A. Categorisation and sample size of 2-minute subsamples per focal male rank; Table B. Observed focal male–rival male pairs and their relative rank; Table C. Model 3: Generalized linear mixed model predicting the presence or absence of a mating gesture by the focal subject, including nearest neighbour variables and visual access to nearest neighbour; Table D. Contingency tables for Models 1–6. (DOCX)
