# Supplementary material for: Gestural Communication and Mating Tactics in Wild Chimpanzees
Source: PLoS One. 2015 Nov 4;10(11):e0139683. doi: 10.1371/journal.pone.0139683 (PMC4633128; doi:10.1371/journal.pone.0139683)
Supplement: S1 File — (DOCX) [file pone.0139683.s001.docx]

**Table A. Categorisation and sample size of 2-minute subsamples per focal male rank**

| **Behavioural category/ focal id (rank)** | | **Nick**  High | **Bwoba**  High | **Musa**  High | **Squibs**  Low | **Kato**  Low | **Hawa**  Low | **Median (IQ range)/ subject** |
| --- | --- | --- | --- | --- | --- | --- | --- | --- |
| Gesture | | | | | | | | |
|  | Present | 1 | 2 | 0 | 2 | 13 | 19 | 2 (0.75 – 14.50) |
|  | Absent | 121 | 55 | 65 | 143 | 233 | 281 | 132 (62.50 – 245) |
| Modality | | | | | | | | |
|  | Visual/ tactile | 1 | 1 | 0 | 2 | 1 | 3 | 1 (0.75 – 2.25) |
|  | Auditory | 0 | 1 | 0 | 0 | 12 | 16 | 0.50 (0 – 13) |
| Copulation | | | | | | | | |
|  | Present | 0 | 2 | 0 | 1 | 3 | 5 | 1.5 (0- 3.5) |
|  | Absent | 1 | 0 | 0 | 0 | 5 | 6 | 0.5 (0 – 5.25) |

**Table B. Observed focal male – rival male pairs and their relative rank**

| Focal male | Rival male |
| --- | --- |
| Rival lower ranking than focal | |
| Bwoba | Kwezi |
| Bwoba | Musa |
| Bwoba | Squibs |
| Hawa | Kwezi |
| Hawa | Zig |
| Kato | Hawa |
| Kato | Kwezi |
| Kato | Zig |
| Musa | Squibs |
| Nick | Bwoba |
| Nick | Kato |
| Nick | Musa |
| Nick | Squibs |
| Nick | Tinka |
| Squibs | Hawa |
| Squibs | Kato |
| Squibs | Simon |
| Squibs | Zalu |
| Rival higher ranking than focal | |
| Bwoba | Nick |
| Hawa | Kato |
| Hawa | Musa |
| Hawa | Nick |
| Hawa | Squibs |
| Hawa | Tinka |
| Hawa | Zefa |
| Kato | Musa |
| Kato | Nick |
| Kato | Zefa |
| Musa | Bwoba |
| Musa | Nick |
| Squibs | Bwoba |
| Squibs | Nick |

**Table C. Model 3:** **Generalized linear mixed model predicting the presence or absence of a mating gesture by the focal subject, including nearest neighbour variables and visual access to nearest neighbour. Overall percentage of cases assigned correct = 93.8%.**

| Predictor variable | Coefficient estimate | Standard error | *t* | p |
| --- | --- | --- | --- | --- |
|  |  |  |  |  |
| *Fixed effects* |  |  |  |  |
| Wind | 15.457 | 0.342 | 45.187 | <0.001*** |
| Oestrus females | -0.159 | 0.505 | -0.314 | 0.754 |
| Rival orientation to focal | 1.560 | 0.524 | 2.979 | 0.003** |
| Nearest neighbour proximity | -0.184 | 0.046 | -4.049 | <0.001*** |
| Nearest neighbour orientation to focal | -1.146 | 0.361 | -3.176 | 0.002** |
| Focal orientation to nearest neighbour | -1.477 | 0.312 | -4.741 | <0.001*** |
| Nearest neighbour visual access | -13.618 | 0.393 | -34.665 | <0.001*** |
| *Covariance parameters* |  |  |  |  |
| Focal male | 0.843 | 1.228 |  | 0.493 |
| Focal male x sample number | 1.077 | 1.006 |  | 0.284 |

*p < 0.05, **p < 0.01, ***p < 0.001

See Table 1 for definition and descriptive data for predictor variables included in this Model.

**Table D. Contingency tables for Models 1 - 6**

a) The observed and predicted fitted values of the number of samples with gestures present and absent (Model 1)

| Observed | Predicted | |
| --- | --- | --- |
|  | Present | Absent |
| Present | 12.9% (4) | 87.1% (27) |
| Absent | 0.1% (1) | 99.9% (816) |

b) The observed and predicted fitted values of the number of samples with gestures present and absent (Model 2)

| Observed | Predicted | |
| --- | --- | --- |
|  | Present | Absent |
| Present | 42.9% (6) | 57.1% (8) |
| Absent | 0% (0) | 100% (146) |

c) The observed and predicted fitted values of the number of samples with gestures present and absent (Model 3)

| Observed | Predicted | |
| --- | --- | --- |
|  | Present | Absent |
| Present | 28.6% (4) | 71.4% (10) |
| Absent | 0% (0) | 100% (146) |

d) The observed and predicted fitted values of number of samples with auditory gestures and visual/ tactile gestures (Model 4)

| Observed | Predicted | |
| --- | --- | --- |
|  | Auditory | Visual/tactile combined |
| Auditory | 100% (24) | 0% (0) |
| Visual/tactile combined | 0% (0) | 100% (7) |

e) The observed and predicted fitted values of number of samples with auditory gestures and visual/ tactile gestures (Model 5)

| Observed | Predicted | |
| --- | --- | --- |
|  | Auditory | Visual/tactile combined |
| Auditory | 100% (7) | 0% (0) |
| Visual/tactile combined | 0% (0) | 100% (7) |

f) The observed and predicted fitted values of the number of samples with copulations present and absent (Model 6)

| Observed | Predicted | |
| --- | --- | --- |
|  | Present | Absent |
| Present | 90% (9) | 10% (1) |
| Absent | 0% (0) | 100% (10) |
